# Supplementary material for: autoFISH: a modular toolbox for sequential single-molecule RNA FISH experiments
Source: Commun Biol. 2026 Apr 6;9:752. doi: 10.1038/s42003-026-09979-z (PMC13230740; doi:10.1038/s42003-026-09979-z)
Supplement: Supplementary file 2 — Supplementary Information [file 42003_2026_9979_MOESM2_ESM.pdf]

Supplementary Information

autoFISH: a modular toolbox for sequential single-molecule RNA FISH experiments

Christian Weber[1,2,*], Thomas Defard[1,2,3,4,5*], Chloé Sturmach[1], Mickael Lelek[6], Hugo Laporte[6,7], Ayan Mallick[6], Maria Isabella Gariboldi[1,2], José-Arturo Londoño-Vallejo[6], Thomas Walter[3,4,5], Charles Fouillade[6], Jacques Bourg[1], Florian Mueller[1,2,*,#]

[1] Institut Pasteur, Université Paris Cité, Photonic Bio-Imaging, Centre de Ressources et Recherches Technologiques (UTechS-PBI, C2RT), Paris, France
[2] Institut Pasteur, Université Paris Cité, Imaging and Modeling Unit, Paris, France
[3] Centre for Computational Biology (CBIO), Mines Paris, PSL University, Paris, France
[4] Institut Curie, PSL University, 75005, Paris, France
[5] INSERM, U900,Paris, France
[6] Institut Curie, Inserm U1021-CNRS UMR 3347, University Paris-Saclay, PSL Research University, Centre Universitaire, Orsay Cedex, France.
[7] Institute of Cell Biology (Cancer Research), University Hospital Essen, Essen, Germany.

Supplementary Figure 1 2
Supplementary Figure 2 3
Supplementary Figure 3 4
Supplementary Note 1 5

Supplementary Figure 1

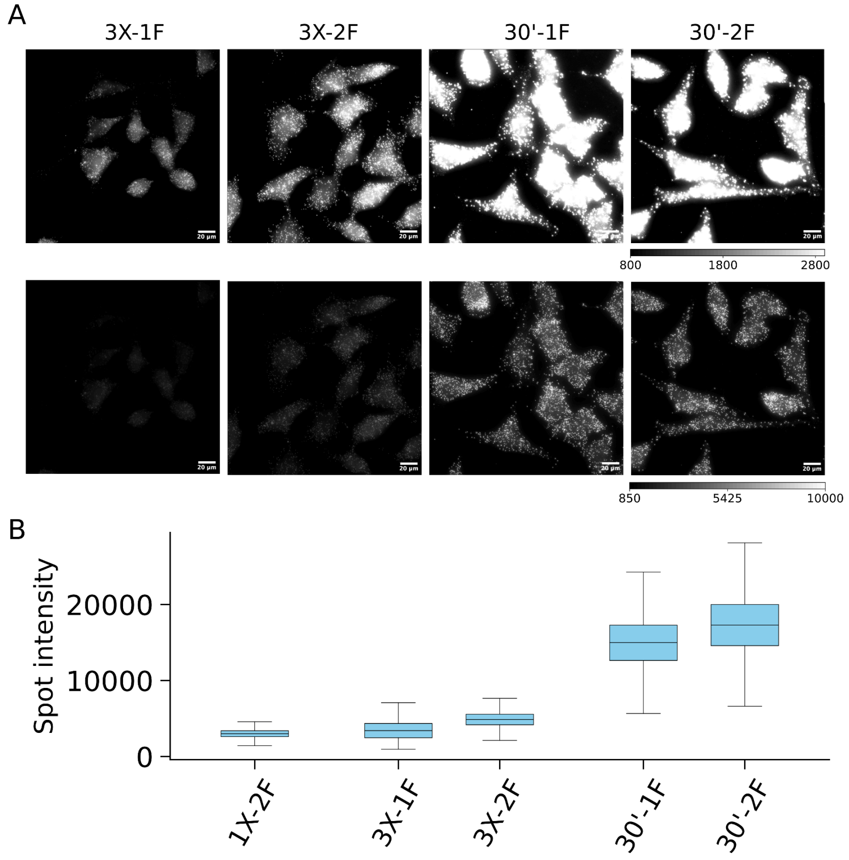

SABER - impact of repeated binding sites and number of fluorophores on single RNA signal intensity

- **1X-2F**: standard FISH with a single binding site; visualized with imager oligos with two fluorophores.
- **3X-1F / 3X-2F**: SABER with three binding sites (ordered from IDT); visualized with imager oligos with either one or two fluorophores.
- **30’-1F / 30’2F**: SABER with enzymatically amplified sequences (30 min for 8-10 binding sites); visualized with imager oligos with either one or two fluorophores.

A Images showing a typical field of view with the same intensity rescaling for each row. The upper row shows a rescaling for the 3X SABER, the lower row for the 30’ SABER. Scale bars are 20 µm.

B Optimization of signal intensity via multivalent and dual-labeled oligonucleotides. Comparison of single RNA spot intensity distributions. Boxplots: central line = median; box = Interquartile range (Q1–Q3); whiskers = max/min data points within 1.5× IQR. Outliers excluded.

Number of fields of view = 3; number of cells: 1X-2F = 23, 3X-1F = 26, 3X-2F = 34, 30’-1F = 29, 30’-2F = 22; number of RNAs: 1X-2F = 2.8K, 3X-1F = 4.5K, 3X-2F = 7K, 30’-1F = 6.9K, 30’-2F = 5.5K.

Supplementary Figure 2

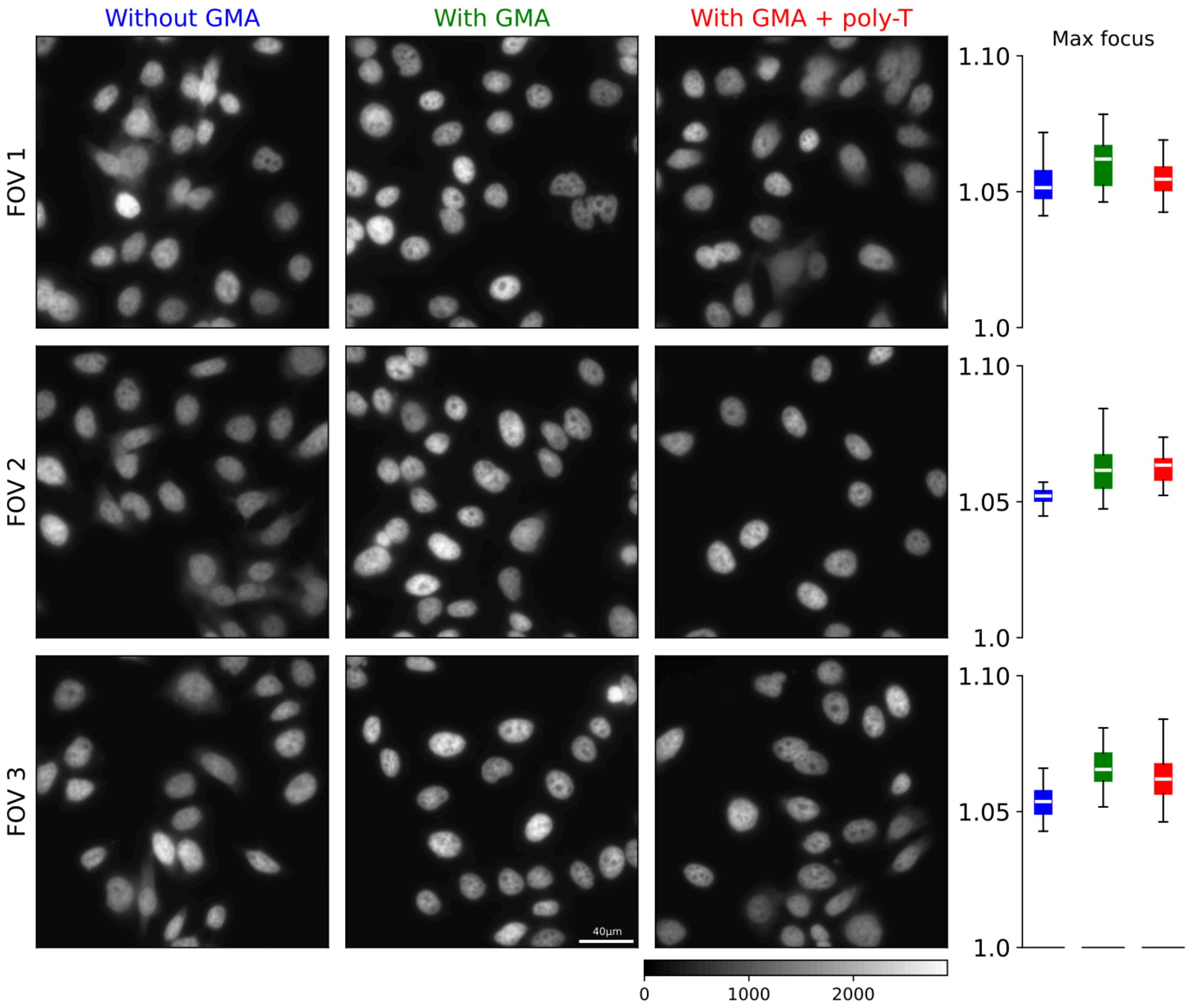

Impact of clearing and anchoring strategy on nuclear DAPI signal.

A Composite images for three different fields of view, without epoxyde (GMA), with epoxyde, and with epoxyde and poly-T anchor oligos. Each nucleus is displayed at the plane in which the average Helmi and Scherer focus metric is maximal. Shared color and spatial scale across all images. Scale bar is 40 μm.

B Maximal mean focus distribution across cells of the field of view. Boxplots: central line = median; box = Interquartile range(Q1–Q3); whiskers = max/min data points within 1.5× IQR. Outliers excluded. Number of fields of view = 9. Number of cells = 266.

Supplementary Figure 3

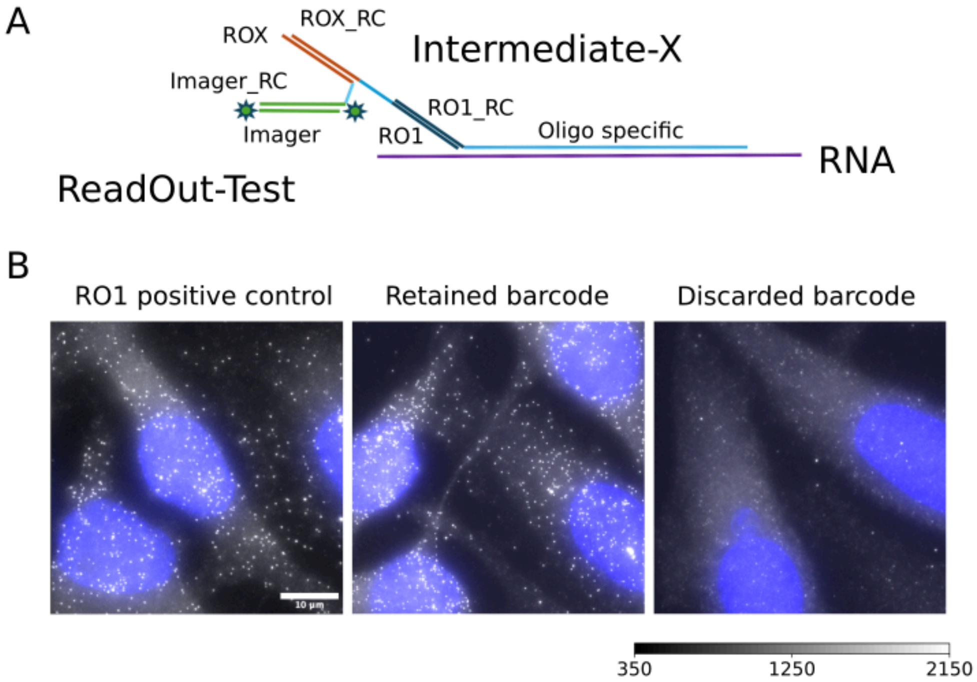

Evaluation of redesigned readout (RO) sequences using intermediary oligonucleotides

A Schematic of the different oligonucleotides used to test new RO sequences. The use of an intermediary oligonucleotide enables the reuse of the same primary-RO oligo pool.
B Comparison of FISH signal intensities used to validate the new RO sequences. Scale bar is 10 µm.

- **First:** Readout 1 without an intermediate oligo to provide a reference image (positive control).
- **Second:** Test of Readout 15 (via intermediary oligo). Image quality is high, barcode is retained.
- **Third:** Test of Readout 16 (via intermediary oligo). Images quality is low, barcode is removed.

Supplementary Note 1

Helmli and Scherer's sharpness metric

For each pixel x,y in a 2-D image, we compute the ratio:

$R(x,y) = I(x,y) / \mu(x,y)$ if $I(x,y) \geq \mu(x,y)$ and $\mu(x,y)/I(x,y)$ otherwise.

$I(x,y)$ is the intensity of the pixel (x,y), and $\mu(x,y)$ is the mean intensity of the pixels in its neighborhood. For a 3D image, we compute this metric for each z-surface.

For a uniform background, R is 1. If a pixel is brighter than the neighborhood's mean, we compute $I(x,y)/\mu(x,y)$, and if it's darker, we compute $\mu(x,y)/I(x,y)$: deviations above or below the mean are treated equally. Therefore, this metric provides a quantitative assessment of local contrast.
