# Supplementary material for: autoFISH: a modular toolbox for sequential single-molecule RNA FISH experiments
Source: Commun Biol. 2026 Apr 6;9:752. doi: 10.1038/s42003-026-09979-z (PMC13230740; doi:10.1038/s42003-026-09979-z)
Supplement: Supplementary file 3 — Description of Additional Supplementary Files [file 42003_2026_9979_MOESM3_ESM.pdf]

Description of Additional Supplementary Files

File name: Supplementary Data 1

Description: This file provides a comprehensive list of all primary probe sequences used for smFISH imaging in this study.
